# An Improved Model of the *Trypanosoma brucei* CTP Synthetase Glutaminase Domain–Acivicin Complex

**DOI:** 10.1002/cmdc.201700118

**Published:** 2017-03-31

**Authors:** Juliana Oliveira de Souza, Alice Dawson, William N. Hunter

**Affiliations:** ^1^ Division of Biological Chemistry and Drug DiscoveryCollege of Life SciencesUniversity of DundeeDundeeDD1 5EHScotlandUK

**Keywords:** acivicin, CTP synthetase, glutaminase, structure-based drug discovery, trypanosomiasis

## Abstract

The natural product acivicin inhibits the glutaminase activity of cytidine triphosphate (CTP) synthetase and is a potent lead compound for drug discovery in the area of neglected tropical diseases, specifically trypanosomaisis. A 2.1‐Å‐resolution crystal structure of the acivicin adduct with the glutaminase domain from *Trypanosoma brucei* CTP synthetase has been deposited in the RCSB Protein Data Bank (PDB) and provides a template for structure‐based approaches to design new inhibitors. However, our assessment of that data identified deficiencies in the model. We now report an improved and corrected inhibitor structure with changes to the chirality at one position, the orientation and covalent structure of the isoxazoline moiety, and the location of a chloride ion in an oxyanion binding site that is exploited during catalysis. The model is now in agreement with established chemical principles and allows an accurate description of molecular recognition of the ligand and the mode of binding in a potentially valuable drug target.

CTP synthetase [EC: 6.3.4.2] catalyzes the formation of CTP from UTP by coupling dephosphorylation of ATP with deamination of glutamine to glutamate, the latter to supply the required amino group. The enzyme, which consists of distinct synthetase and glutaminase domains, is rate limiting in the synthesis of cytosine nucleotides required to maintain RNA and DNA levels. Given such a critical role in metabolism CTP synthetase represents a potential target for therapeutic intervention in a range of diseases.^1^, ^2^ This extends to trypanosomiasis, or African sleeping sickness, a potentially fatal infection with the protozoan parasite *Trypanosoma brucei*. This parasite possesses a low level of CTP and moreover is unable to salvage cytosine from the human host, suggesting the enzyme as a favorable point of intervention.^2^, ^3^, ^4^ We assessed the potential of this target for early stage drug discovery in trypanosomaisis. A major consideration is whether such a target is enabled with access to structural information.^5^ In this case, the Structural Genomics Consortium (SGC, www.thesgc.org) had determined the structure of the *T. brucei* glutaminase domain in complex with acivicin, deposited coordinates and structure factors in the RCSB Protein Data Bank (PDB) in 2008 (PDB ID: 2W7T). Acivicin (Figure 1), a fermentation product of *Streptomyces sviceus,* inhibits enzymes like CTP synthetase that catalyze amido transfers from l‐glutamine. This natural product displays potent anticancer activities, however, it has not progressed beyond phase 1 clinical trials due to neurotoxicity.^6^ Nevertheless, the compound displays antitrypanosomatid activity and as such the structure of a CTP synthetase complex with a lead compound is potentially valuable. Indeed, the SGC model has been used for docking calculations which formed the basis for studies reported in *ChemMedChem* where researchers sought to design acivicin analogues as more potent *T. brucei* CTP synthetase inhibitors.^7^

**Figure 1:**
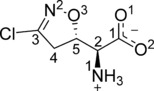
The structure and numbering scheme of acivicin, (2*S*)‐amino[(5*S*)‐3‐chloro‐4,5‐dihydro‐1,2‐oxazol‐5‐yl]ethanoic acid.

However, our inspection of the available data and the SGC model revealed several issues. The details in the drug binding site were inconsistent with the structure of acivicin (Figure 1) in terms of the chiral center at C5, the hybridization at C3, some covalent bond lengths and the hydrogen bonding capacity of the ligand was not optimized. Moreover, difference Fourier syntheses, electron and difference density including omit maps suggested the orientation of the isoxazoline ring was incorrect (Supporting Information Figures S1–S3). We extended the crystallographic refinement seeking to address these deficiencies and now present a model consistent with established chemical principles.

Coordinates and structure factor data for PDB ID: 2W7T were inspected and compared with those output from PDB‐REDO, an automated refinement process designed to improve crystallographic models.^8^ In common with our experience,^9^ the PDB‐REDO model was identified as being significantly improved, and provided the starting point for further refinement. The inspection of electron and difference density maps and model manipulation were carried out using COOT^10^ with least‐squares calculations performed in REFMAC5.^11^ Water molecules, three chloride ions and several side chain conformers were included in the model. The dictionary of ligand restraints was assembled using *Grade*.^12^ Geometry was assessed with MolProbity^13^ and the PDB Validate Tools. Figures were generated with PyMOL (Schrödinger). Coordinates have been deposited with the PDB (PDB ID: 5N29).

The glutaminase domain of *T. brucei* CTP synthetase, (residues 319–589), following incubation with acivicin, crystallized in space group *P*2_1_2_1_2_1_ with one molecule in the asymmetric unit and diffracted to 2.1 Å resolution. Three refined models are available, the original PDB entry (PDB ID: 2W7T), the PDB‐REDO version and from this current study, with crystallographic statistics collated and compared as Supporting Information Table S1. Our continued refinement produced a model with acceptable geometric parameters and agreement with the diffraction data. This model is essentially the same as from PDB‐REDO with the major difference that the ligand now has correct stereochemistry. This means that the features in the active site relevant to activation, specificity and interactions with the inhibitor can now be accurately described. Of note also is the identification of a chloride ion bound in the active site close to the acivicin adduct. A water molecule with a low isotropic thermal parameter (*B*‐factor ∼5 Å^2^), significantly less than the surrounding residues previously occupied this position. Comparing peak heights in difference density omit maps of S and carbonyl O atoms with this site suggested that it was occupied by some species with more electrons than O, slightly less than S. The site interacts with three amide groups accepting hydrogen bonds of length 3.1–3.2 Å (Gln423, Arg500, Tyr501), a water molecule (3.9 Å) and 3.6 Å distant from acivicin C4. We therefore included chloride at this position (see below, *B*‐factor 24.3 Å^2^), the ion likely derived from the crystallization and cryo‐protectant conditions, which included 300 mm NaCl. We refined the ion at full occupancy but recognize the possibility that it is a mixed water/ion site. The density maps at Phe393 do not match to the size and shape of that side chain being more suggestive of a methionine or leucine. Without recourse to sequence information on the expression system we left this as a phenylalanine, which is consistent with genomic data.

The glutaminase domain is dominated by a core β‐sheet of seven strands decorated on one side by six α‐helices, on the other by four helices and two parallel β‐strands (Figure 2). The active site is located at one end of the sheet in a small, ordered, polar cavity. One side of this cavity is lined by two polypeptide segments extending from the pair of strands, the other by a C‐terminal extension to a β‐strand that is part of the core sheet. The width of the cavity is about 8 Å as measured from Gly391 N to Arg498 O, this is a vector that bisects the space between the ligand C1 and Cys419 S atoms.

**Figure 2:**
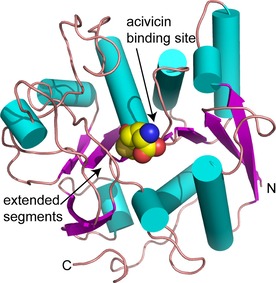
Secondary structure, fold, and acivicin binding site in the glutaminase domain of *T. brucei* CTP synthetase. Helices are shown as cyan cylinders, β‐strands as purple arrows, and the polypeptide in extended conformation as a brown coil. The covalent modification following reaction with acivicin is depicted as van der Waals spheres (C: yellow, N: blue, O: red, S: orange). The positions of the N‐ and C‐terminal residues of the domain are labeled.

The corrected orientation of the ligand now results in four out of five functional groups participating in hydrogen bonding interactions directly with the enzyme, the fifth to a water molecule that is then in contact with the enzyme (Figure 3). N2 and O3 accept hydrogen bonds donated by the main chain amides of Leu420 and Gly392 respectively. The C1 carboxylate interacts with solvent, and the side chains of basic residues Arg498 and His549. The proximity of the Arg498 carbonyl group (3.0 Å) suggests that the carboxylate is protonated. The amino substituent on C2 donates hydrogen bonds to water and the carbonyl of Gly392.

**Figure 3:**
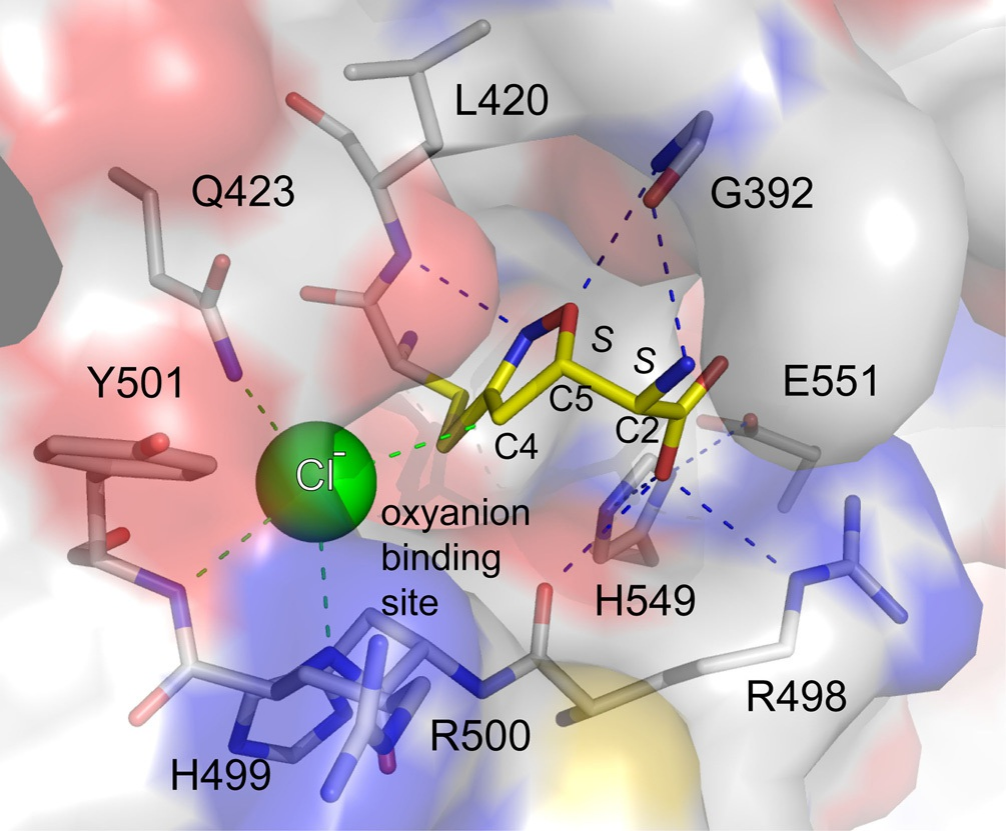
Binding mode of the acivicin–glutaminase domain adduct. The enzyme surface is depicted as a semi‐transparent van der Waals surface, with key residues shown as sticks using the color scheme in Figure 2, except protein C atoms are colored gray. Potential hydrogen bonds are depicted as dashed lines. The hydrogen bonding interactions involving the acivicin adduct all fall in the range 3.0–3.2 Å. The four dashed lines colored green identify interactions with the chloride ion (green sphere). These are in the range of 3.0–3.2 Å for interactions with amide nitrogen atoms, and we note the potential for a C4‐H⋅⋅⋅Cl^−^ association, distance 3.6 Å. The *S* stereochemistry positions are labeled. For the purpose of clarity, water molecules are not shown.

Although the fit of the isoxazoline moiety to the electron density is supportive of sp^2^ hybridization at C3, at 2.1 Å resolution the data are insufficient to provide certainty in this respect. However, inspection of the electron density associated with the high resolution 1.5 Å structure of *Helicobacter pylori* γ‐glutamyltranspeptidase is unambiguous in the assignment of an sp^2^ C3.^14^, ^15^ This would be consistent with our refined model and supports a straightforward mechanism of reaction whereby acivicin undergoes nucleophilic attack from Cys419, leading to the formation of a tetrahedral oxyanion with sp^3^‐hybridized C3, then a collapse of this intermediate with release of chloride and restoration of the starting point sp^2^ C3 and covalent linkage to Cys419. The assignment of a C3=N2 double bond is further supported by the hydrogen bonding interaction whereby the Leu420 amide donates to the acceptor N2. We note also that an sp^2^‐hybridized C3 is assigned in the high‐resolution structure of *Bacillus subtilis* γ‐glutamyltranspeptidase.^16^ In stark contrast an sp^3^‐hybridized C3 is reported in the structure of the *Escherichia coli* γ‐glutamyltranspeptidase acivicin adduct.^17^ However, in this case the difference Fourier synthesis based on PDB ID: 2Z8K for this structure (not shown) presents significant positive and negative features that suggest deficiencies in the model. Moreover, the authors invoke a highly complicated mechanism that involves acivicin ring opening followed by ring closure to leave an anionic N2 group. We judge that this is unlikely and that established chemical principles explain the formation of the covalent adduct with sp^2^‐hybridized C3 as noted above.

The activation of the nucleophilic Cys419 is supported by the position of His549, 3.6 Å distant, which in turn is positioned by a hydrogen bond with the side chain of Glu551. Although His499 is nearby and an alternative rotamer could position the basic side chain close to the cysteine thiol, we note that a hydrogen bond with Glu502 (not shown) helps to select for the observed rotamer holding the basic side chain away from the active site. Stabilization of the oxyanion intermediate formed during the reaction with acivicin, or during catalysis may benefit from the position of the amine and amide groups of Gln423, Arg500 and Tyr501 respectively forming a positively charged environment similar to that observed near the cysteine protease‐like catalytic triad in trypanothione synthetase‐amidase.^18^ This site is where chloride binds.

The PDB is a hugely valuable resource for biochemical and medicinal chemistry research but unfortunately, serious errors in ligand–protein complexes are not uncommon.^8^ For our own part, we previously identified the incorrect structure of the potent antifolate and anticancer agent assigned as LY374571,^19^ and showed that structures of the fatty acid binding site of the human peroxisome proliferator‐activated receptors‐β/δ are not occupied by lipid‐lowering synthetic agents but rather by endogenous ligands derived from the bacterial expression system.^20^ In respect of the glutaminase domain of CTP synthetase from *T. brucei*, then our inspection identified deficiencies in the crystallographic model. These have been corrected and future efforts to obtain novel lead compounds might progress with an accurate template of the binding site occupied by an inhibitor and an anion now available.

## Conflict of interest

*The authors declare no conflict of interest*.

## Supporting information

As a service to our authors and readers, this journal provides supporting information supplied by the authors. Such materials are peer reviewed and may be re‐organized for online delivery, but are not copy‐edited or typeset. Technical support issues arising from supporting information (other than missing files) should be addressed to the authors.

Supplementary
