# Supplementary material for: An Improved Model of the Trypanosoma brucei CTP Synthetase Glutaminase Domain–Acivicin Complex
Source: ChemMedChem. 2017 Mar 31;12(8):577–9. doi: 10.1002/cmdc.201700118 (PMC5413811; doi:10.1002/cmdc.201700118)
Supplement: Supplementary file 1 — Supplementary [file CMDC-12-577-s001.pdf]

CHEMMEDCHEM
Chemistry & Drug Discovery

Supporting Information

An Improved Model of the *Trypanosoma brucei* CTP Synthetase Glutaminase Domain–Acivicin Complex

Juliana Oliveira de Souza, Alice Dawson, and William N. Hunter*[a]

cmdc_201700118_sm_miscellaneous_information.pdf

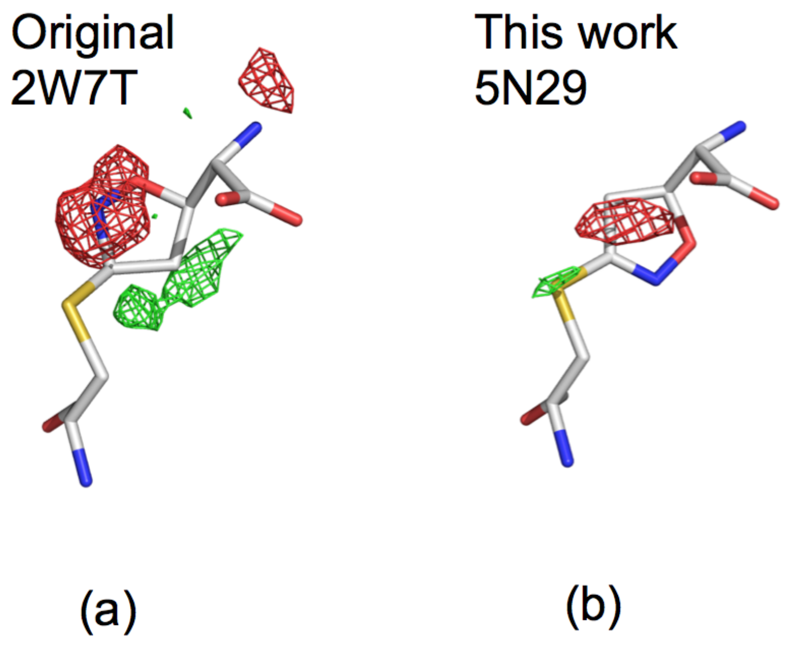

Figure S1. Difference density Fourier syntheses. (a) Calculated on the basis of phases from the original PDB entry 2W7T and (b) based on the model following further refinement and alteration of chirality at C5. The red mesh represents a -2.5σ contour level and the green mesh +2.5σ.

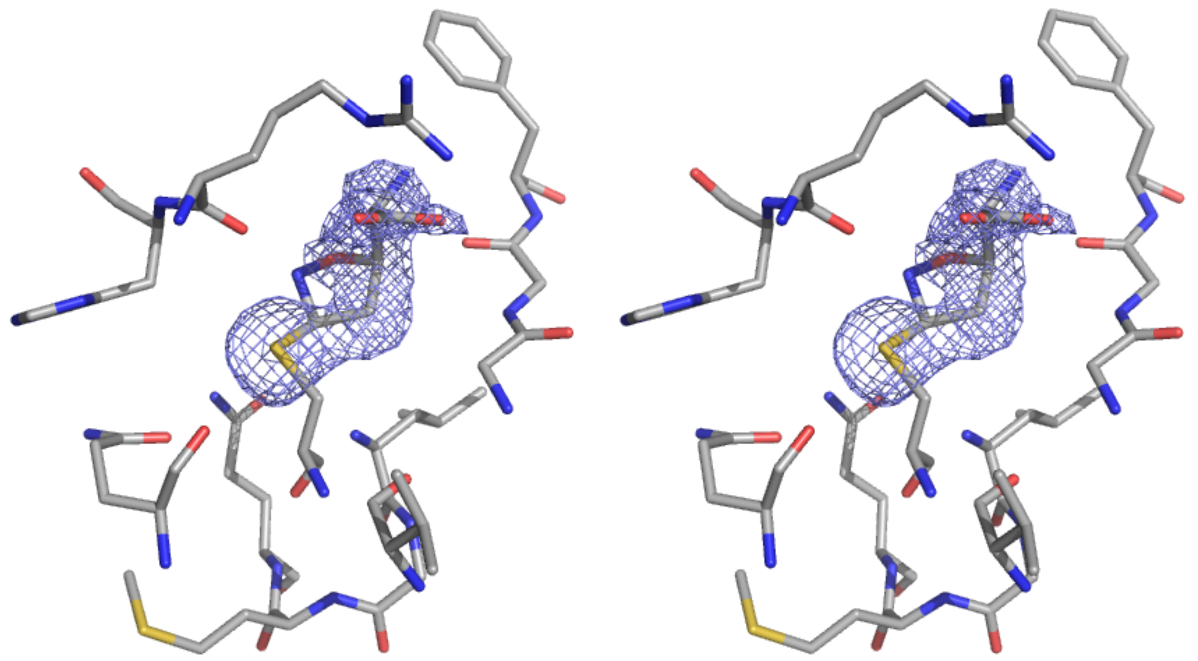

Figure S2. Stereoview into the active site showing the omit Fo-Fc map (blue chicken wire) contoured at 3σ for the acivicin adduct in PDB entry 2W7TQ. A similar orientation to Figure S1 is used.

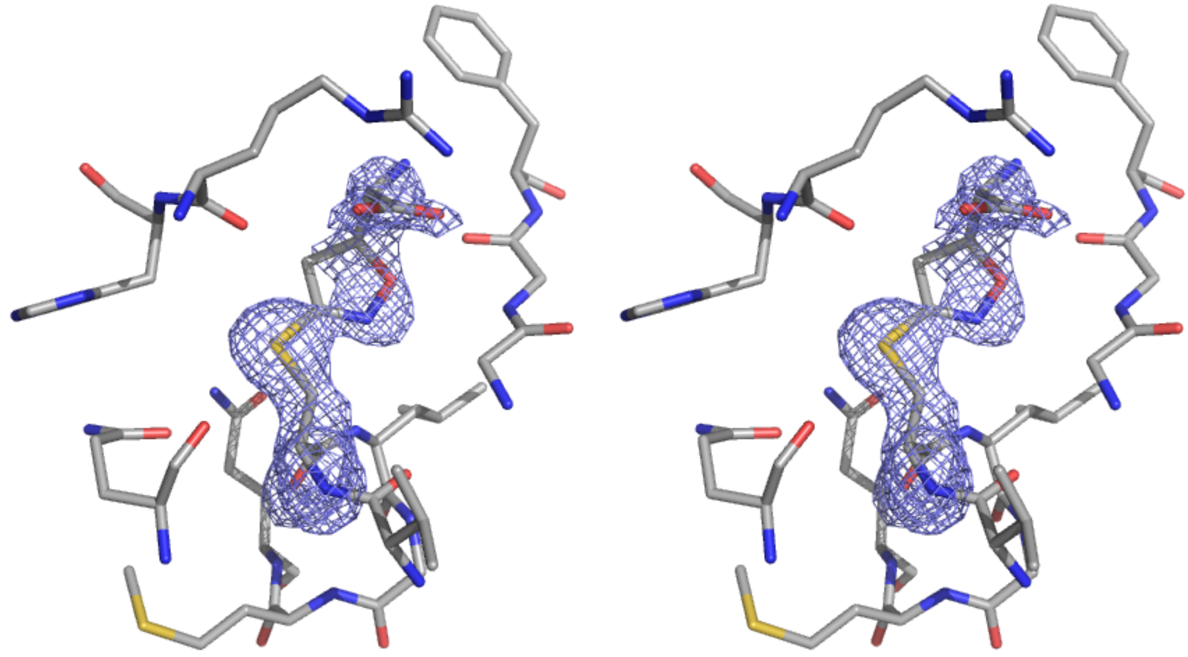

Figure S3. Stereoview showing the omit Fo-Fc map (blue chicken wire) contoured at 3σ for the further refined acivicin adduct presented in this work. A similar orientation to Figure S1 is used.

.

Table S1. Crystallographic statistics.

**Structure**	*PDB*	*PDB-REDO*	*This work*
**PDB codes**	2W7T	2W7T	5N29
**Data collection and Processing**			
Space group, unit cell lengths<br>*a, b, c* [Å]	$P2_12_12_1$	50.48, 63.68,	76.58
Resolution ranges [Å]	19.78 – 2.10		
Unique reflections, redundancy	14866, 7.8		
R_{merge}[a]	0.08 (0.22)		
Wilson *B* [Å^2]	17.3		
Completeness [%],<I/σ(I)>	99.1, 4.1		
**Refinement**			
R values (R_{work}/R_{free})[b] [%]	19.9 / 26.2	19.35 / 23.20	19.34 / 23.53
Number reflections for R_{work}/R_{free}	14121 / 744	14087 / 740	14087 / 740
Protein residues	264	264	265
Anions	2 Cl^-	2 Cl^-	3 Cl^-
Other atoms (acivicin adduct)	10	10	10
Water molecules	99	96	126
**RMSDs**			
Bonds [Å]	0.012	0.013	0.008
Angles [°]	1.254	1.60	1.23

**Ramachandran plot**			
Residues in favored regions [%]	97.3 (252)	98.1 (254)	98.1 (255)
Residues in allowed regions [%]	7	5	5
Residues in outlier regions [%]	0	0	0
**Mean B-factors**			
Protein atoms [Å^2]	17.03	12.23	19.11
Water molecules [Å^2]	20.18	22.34	23.45
Ions [Å^2]	20.69	23.35	23.38
Ligand [Å^2]	20.96	22.87	21.41

*Numbers in parenthesis are for the highest resolution shell. Values in parentheses correspond to the highest resolution shell. [a.] $R_{merge} = \sum h \sum i||(h,i)$ - <I(h)> $\sum h \sum i$ I(h,i); where I(h,i) is the intensity of the ith measurement of reflection h and <I(h)> is the mean value of I(h,i) for all i measurements. [b.] $R_{work} = \sum hkl||F_o|-|F_c||/\sum|F_o|$, where F_o is the observed structure factor amplitude and the F_c is the structure-factor amplitude calculated from the model. R_{free} is calculated with a subset of data that are excluded from refinement calculations (5 %) using the same method as for R_{merge}.
